# Quality employee–manager relationships are key to career success

**DOI:** 10.1177/13623613251370814

**Published:** 2025-09-01

**Authors:** Susan M Hayward, Sophie Hennekam, Simon M Bury

**Affiliations:** 1 La Trobe University, Australia; 2 Audencia Business School, France

**Keywords:** growth, leader, LMX, neurodiversity, supervisor

## Abstract

This study investigated the relationship between the perceived quality of employee–manager relationships and workplace outcomes, and whether these differed between autistic and non-autistic employees. We surveyed 189 employed participants (*n* = 92 autistic, *n* = 97 non-autistic) from the United Kingdom. Participants completed measures of employee–manager relationship quality; workplace behaviours, for example, strengths use and job crafting; and outcomes, for example, career development opportunities and job satisfaction. Multiple regression analyses assessed the association between employee–manager relationship and autism diagnosis on behaviours and outcomes. One’s relationship with their manager was a significant predictor of all workplace behaviours and outcomes, explaining 22% of the variance in career development opportunities and 7%–11% for other dependent variables. Autism diagnosis was not an independent predictor of the quality of the employee–manager relationship and outcomes. Autistic participants rated the relationship with their managers as poorer than non-autistic employees. This study emphasises the importance of positive employee–manager relationships for career success, irrespective of neurotype. However, the poorer ratings of these relationships by autistic employees highlight a critical need for strategies to enhance employee–manager relationships.

**Lay Abstract:**

**Quality Employee–Manager Relationships are Key to Career Success**

***Why is this an important issue?***

Finding and keeping meaningful work is challenging for many autistic adults. A key factor in career success is the relationship between employees and their managers. Research shows that a strong relationship with one’s manager can help employees use their strengths, feel more satisfied in their jobs and grow in their careers. However, little is known about how this applies to autistic employees, who may face unique challenges in the workplace. Understanding how these relationships affect autistic employees is important for creating supportive, inclusive and successful work environments.

***What was the purpose of this study?***

The study aimed to explore whether the quality of the relationship between employees and their managers affects autistic employees’ workplace success. The researchers wanted to know if this relationship impacts things like job satisfaction, career development opportunities and the ability to use their strengths at work. They also compared autistic employees to non-autistic employees to see if there were any differences in these areas.

***What did the researchers do?***

The researchers surveyed 189 employees from the United Kingdom, including 92 autistic and 97 non-autistic participants. They asked participants about their relationship with their manager, how often they used their strengths at work, their job satisfaction and their career development opportunities. The researchers analysed this data to see how much the quality of the manager–employee relationship influenced workplace outcomes.

***What were the results and conclusions of the study?***

The study found that the quality of the relationship with one’s manager was a strong predictor of workplace success for both autistic and non-autistic employees. This relationship was more important than whether an employee was autistic. However, autistic participants rated their relationships with their managers as poorer than non-autistic participants.

Achieving job–person–environment fit is often cited as a solution to autism employment issues (Hayward & Flower, 2023; Hayward et al., 2019a). Research on job–person–environment fit often emphasises the role of organisational factors as drivers of employment success (Xu et al., 2023). However, a critical but frequently overlooked element of fit is the relationship between an employee and their direct supervisor, or manager. For the general population, a positive relationship with one’s manager is associated with career success (Ali & Anwar, 2021; Mumtaz & Rowley, 2020). Such relationships enable employees to tailor their job to meet their needs and use their strengths, as well as improve career progression opportunities and job satisfaction (Mumtaz & Rowley, 2020; Xu et al., 2023).

Emerging research indicates that for autistic employees, the relationship with their manager is similarly, if not critically, important for successful employment (Hayward et al., 2020; Martin et al., 2023). To illustrate, Hayward et al. (2020) determined that having a positive relationship with one’s manager was equally endorsed by autistic and non-autistic employees as an important support to help them manage workplace pressures. Martin et al. (2023) later found that high-quality employee–-manager relationships significantly contributed to job satisfaction and retention among autistic employees. Yet, more specific and explicit impact of managers on the career success of autistic people remains underexplored (Raymaker et al., 2023). For example, no determination has yet been made as to if a positive relationship with one’s manager directly impacts autistic employees’ ability to utilise their strengths or progress their career. Understanding these mechanisms is essential for informing manager training and development initiatives to create autism inclusive work environments, supporting autistic employees to improve their job fit.

Such insights are particularly urgent given the significant underrepresentation of autistic people in employment and the unique barriers they face particularly in relation to social communication (Bury et al., 2021; Bury, Hedley, et al., 2024; Hayward et al., 2022). Autistic people’s career paths are often marked by negative experiences, frequent job transitions (Hayward et al., 2018a, 2019a) and underutilisation of their skills (Davies et al., 2024). These experiences often lead to unfulfilling roles that fail to leverage their full skill set and potential (Hayward et al., 2019b). In the absence of supportive and inclusive work environments, autistic employees face limited opportunities for professional growth, perpetuating cycles of underutilisation and career stagnation (Davies et al., 2024).

In the general population, a supportive manager can foster an environment where employees feel empowered to utilise their strengths (Audenaert et al., 2020; Huyghebaert-Zouaghi et al., 2021). When managers encourage open communication and collaboration, employees are more likely to express their preferences and aspirations regarding their work (Martin & Harrison, 2022). This mutual understanding allows for adjustments that enhance both individual satisfaction and organisational effectiveness (Hoff et al., 2020; Tims et al., 2015).

For autistic employees, job crafting, whereby employees actively shape their roles to better align with their skills, interests and values, is a potential avenue for achieving better job fit (Wrzesniewski & Dutton, 2001; Wrzesniewski et al., 2013). However, research reveals lower job crafting engagement among individuals with disabilities (Brucker & Sundar, 2020). Although participation in job crafting is yet to be explored in an autistic population, this disparity is significant given job crafting is linked to higher levels of job satisfaction (J. Li et al., 2023). In addition, for underutilised employees, job crafting can leverage the extent to which one uses their strengths and therefore achieve better job fit (Zhang et al., 2021). Crafting can also be a way of obtaining workplace adjustments without necessarily disclosing a diagnosis of autism.

The quality of the employee–manager relationship is also associated with job satisfaction (Stringer, 2006). When employees feel valued and understood by their managers, they are more likely to experience a sense of belonging within the organisation (Randel et al., 2018). This connection not only enhances daily work experiences but also contributes to employment sustainability (Blau et al., 2023). Sustainable employment is vital for fostering long-term career development (Van der Heijden et al., 2020).

Managers play a pivotal role in career progression by actively guiding employees towards professional growth opportunities that align with their skills and career goals. Through avenues such as promotions, training programmes, mentorship and involvement in special projects, managers can help employees build competencies, gain new experiences and expand their professional networks (Bozionelos et al., 2020; Cheng et al., 2024). Access to these career development opportunities is fundamental to career success. As such, effective managerial support and investment in employees’ growth engender greater opportunities for career sustainability and advancement.

Thus, our aim was to examine the impact or relative importance of the employee–manager relationship for autistic people’s career. Specifically, we investigated the degree to which the relationship with one’s manager, alongside the experience of being autistic, is predictive of workplace behaviours and outcomes. These were using one’s strengths at work, participation in job crafting behaviours, the perception of career development opportunities, and job satisfaction.

## Method

### Participants

Participants were 189 individuals (autistic, *n* = 92; non-autistic, *n* = 98; *M_age_* *=* 36.59 years; *SD* = 10.09) employed by a third party (i.e. not self-employed) in the United Kingdom. Eligibility criteria for both the autistic and non-autistic groups included being aged 18 or older, having current or past employment experience and residing in the United Kingdom. Participants identifying as autistic were asked whether they had a formal autism diagnosis. Those who confirmed having an autism diagnosis were then asked to specify the age at which they received it to be included in this group (see Table 1). Specific data on socioeconomic status and race/ethnicity were not recorded. See Table 1 for participant demographic information.

**Table 1.** Participant demographic information.

	Autistic *n* = 92; 48%	Non-autistic *n* = 98; 52%
**Gender**		
Women	42; 46%	56; 57%
Men	50; 54%	42; 43%
**Age**	M = 35.97; SD = 10.24	M = 37.17; SD = 9.97
**Age of autism diagnosis (years)**	M = 25.30; SD = 11.80	N/A
	Median = 25.00 years	
	Range 3–59 years	
**Education**		
Non-completion of secondary education*	1; 1%	9; 9%
Secondary education	19; 20%	26; 27%
Diploma or further education college	4; 4%	1; 1%
Degree	43; 47%	35; 36%
Graduate certificate or diploma	1; 1%	5; 5%
Honours	2; 2%	2; 2%
Masters	18; 19%	17; 17%
Doctorate	4; 4%	3; 3%
**Employment status**		
Permanent	86; 94%	89; 91%
Contracted	6; 7%	9; 9%
Hours worked per week*	M = 36.76; SD = 9.09	M = 33.44; SD = 9.73
**International Labour Organization’s (ILO) International Standard Classification of Occupations**	*n* = 91 M = 2.92; SD = 0.73	*n* = 96 M = 2.75; SD = 0.68

* Significant difference between groups, *p* < 0.05.

Participants responded to an online survey consisting of quantitative and qualitative questions. They were not matched on demographic variables. However, autistic and non-autistic participants did not significantly differ on age (*t* = −0.82, *p* = 0.41). There were also no significant differences between groups of participants on their highest level of educational attainment, with the exception that more autistic than non-autistic people completed only secondary education (1% vs 9%, respectively; χ^2^ (1, 189) = 6.24, *p* = 0.01). Autistic people also reported regularly working significantly more hours per week than non-autistic people (*t* = 2.42, *p* = 0.01).

All participants were asked the type of work they currently do, or previously did if not currently employed, as well as their job tasks. They were employed in a wide range of jobs, example responses include:

I repair watches, ensure quality work comes from the other watchmakers. I also do the training for staff with regards to the watches and see customers when required.

I work as a singing teacher in a small private music school. I see students one on one for half an hour and sometimes hour-long lessons. Each lesson will be tailor made to the individual student as I teach many different ages and abilities. We will practice warmups and then performance and theory of music.

I am a line manager for 10 reports working in the nuclear industry including data analysis and report writing as well as software development.

I am a shift manager at a pub running the day-to-day shifts and all administrative work including leading a team of up to 40 people.

I am a sustainability consultant responsible for assisting a team of architects, engineers, ecologists etc. to meet sustainability goals to achieve certification for commercial buildings. I also carry out energy assessments for building regulations compliance.

The participants’ responses regarding work type were compared to the International Labour Organization’s (ILO) International Standard Classification of Occupations. Using each participant’s description of their job role and tasks performed, two independent researchers assigned a skill level ranging from 1 (low) to 4 (high). The researchers agreed on the classification of 94% of cases. In instances of disagreement, they met to discuss and reach a consensus on the appropriate skill level. For those who provided sufficient information for their job role to be classified by skill level (*n* = 187), no statistically significant difference in ILO skill level was found between the autistic and non-autistic samples (*t* (185) = 1.67, *p* = 0.10).

### Method

#### Ethical considerations

After University ethics approval (HREC # 2022-24659-35390-4), the survey to recruit both autistic and non-autistic participants was initially advertised via the researcher’s professional networks. When this method did not generate many organic responses (*N* = 1), the researchers utilised a paid survey platform to recruit all participants, Prolific. Participants recruited via both methods were combined into a single data set.

#### Consent to participate

Completion of the survey was taken as consent to participate in the study. It was stressed to participants that their participation was voluntary, anonymous and they could withdraw from the study at any time.

#### Sample size

To calculate the sample size needed to examine two predictor variables on a single dependent variable with an alpha of 0.01, a priori power analyses using G*Power (Faul et al., 2007) was undertaken. Power analysis determined that a sample of 184–188 participants was required for regression analyses to detect a medium effect size (*f*^2^ = 0.15).

### Materials

The survey consisted of a range of previously validated scales and some demographic questions. After responding to demographic questions (e.g. age, gender), participants answered the main variables of interest, as detailed following.

#### Relationship with manager

Participants’ relationship with their manager (RWM) was obtained with a single open text question, ‘What is the relationship like with your current supervisor?’ These qualitative responses were quantified (Scherp, 2013) to reflect a 5-point Likert-type scale from 1 = *negative* to 5 = *positive*; see ‘Analysis’ section for further information. Two independent coders rated participants’ responses; inter-rater reliability was high, κ = 0.90. Where raters differed, they discussed and agreed on the placement of the response.

#### Strengths use

To measure the degree to which participants believe their individual strengths are utilised in their job, a single subscale from the Strengths Use and Deficit Correction questionnaire was utilised (Van Woerkom et al., 2016). The subscale was the Strengths Use Behaviour, a six-item subscale which concerns the proactive steps employees take to apply their strengths in the workplace. Cronbach’s alpha in the current sample overall, as well as in the autistic and non-autistic sample, was excellent (0.94, 0.92, 0.95, respectively).

#### Job crafting

Job crafting was measured using the 21-item Job Crafting measure derived from the Job Demands-Resources model (Tims et al., 2013). It measures the degree to which employees engage in activities which aim to balance their job resources and demands to meet their own needs (Tims et al., 2012). In the current sample overall, as well as in the autistic and non-autistic samples, Cronbach’s alpha was excellent (0.90, 0.89, 0.91, respectively).

#### Career development opportunities

As there was no single measure that adequately measured the perception of career development opportunities, this study used selected Human Resource Practice scale items from both Armstrong-Stassen (2008) and Saba and Guerin (2005). All questions were rated by participants on a 5-point Likert-type scale from 1 = *never* to 5 = *always*. The questions asked were, ‘Please indicate how often your organisation offers or supports you to have the following’: ‘the opportunity to develop new skills and knowledge’, ‘coaching that supports my development’, ‘good career prospects’ and ‘the possibility to transfer to a job that better suits my needs’. For these items, Cronbach’s alpha in the current sample overall, as well as in the autistic and non-autistic sample, was good (0.84, 0.82, 0.86, respectively).

#### Job satisfaction

Job satisfaction was measured using a single question, ‘Overall, how satisfied or dissatisfied are you with your job?’. This was rated by participants on a 7-point Likert-type scale from 1 = *extremely dissatisfied* to 7 = *extremely satisfied*. We used a single-item scale as research has shown that single-item measures of job satisfaction are easier, take less time to complete and may contain more face validity compared to multi-item scales (Nagy, 2002).

### Analysis

#### Deductive thematic analysis

Deductive thematic analysis (DTA) takes a top-down approach to data analysis where researchers begin by identifying predefined themes or codes based on an existing theory or model (Proudfoot, 2023). In this instance, a model of data categorisation for RWM was created by the authors which was based on a 5-point Likert-type scale: 1 = *negative*, 2 = *slightly negative*, 3 = *neutral*, 4 = *slightly positive* and 5 = *positive*. After the model was created, it was then applied to categorise the data. Responses regarding RWM were used for the sole purpose of quantification using DTA and coded using this codebook.

#### Regression

Multiple regression was used to examine the relationship between each dependent variable, strength use, job crafting, work satisfaction and career development opportunities. Autism diagnosis and RWM were entered as predictor variables into each regression. An interaction term of autism diagnosis and RWM was entered on the second step; however, we acknowledge that sample size may limit the reliability of interaction effects. A conservative alpha of 0.01 was applied owing to multiple comparisons on the same data set.

## Results

### Descriptives

Autistic participants were significantly more likely to rate their RWM lower than non-autistic participants (autistic: *M* = 4.23; *SD* = 1.10, non-autistic: *M* = 4.57; *SD* = 0.76; *t* = −2.51, *p* = 0.01, *d* = –0.37, 95% CI: [–0.65, –0.08]). Example responses from the DTA include: 1 = *negative*, ‘She thinks it is positive, but from my position it is negative’; 2 = *slightly negative*, ‘It is a slightly negative relationship where I am always walking on eggshells’; 3 = *neutral*, ‘It is okay – not that positive as I don’t feel they [my manager] really understand me but I get on enough with them on a professional level to do my job’; 4 = *slightly positive*, ‘. . . I usually feel listened to and appreciated’; 5 = *positive*, ‘Very good and trusting’.

There were no significant differences between participant groups on scores for: use of strengths (autistic: *M* = 34.13; *SD* = 5.32, non-autistic: *M* = 32.86; *SD* = 6.06, *t* = 1.54, *p* = 0.06), job crafting (autistic: *M* = 66.46; *SD* = 14.13, non-autistic: *M* = 63.27; *SD* = 14.53, *t* = 1.53, *p* = 0.06), or career development (autistic: *M* = 12.68; *SD* = 3.81, non-autistic: *M* = 12.36; *SD* = 3.73, *t* = 0.60, *p* = 0.55). However, autistic participants scored significantly lower than non-autistic participants on job satisfaction (autistic: *M* = 4.36; *SD* = 1.70, non-autistic: *M* = 4.85; *SD* = 1.37, *t* = 2.18, *p* = 0.02, *d* = –0.32, 95% CI = [–0.60, –0.03]).

### Regression

Correlations between all variables to verify their independence were examined (see Table 2). There were no correlations between variables above 0.8. However, there was a significant moderate to strong positive correlation between job crafting and career development (*r* = 0.58), and a significant moderate correlation between job crafting and strength use (*r* = 0.52).

**Table 2.** Correlation matrix.

	Autism diagnosis	RWM	Job crafting	Strength use	Career development	Job satisfaction
Autism diagnosis	1	0.18	−0.11	−0.11	−0.04	0.16
RWM	–	1	0.30*	0.24*	0.46*	0.33*
Job crafting	–	–	1	0.52*	0.58*	0.17
Strength use	–	–	–	1	0.44*	0.33*
Career development	–	–	–	–	1	0.39*
Job satisfaction	–	–	–	–	–	1

* Correlation is significant at the *p* = 0.01 level (two-tailed).

RWM individually and significantly contributed to each model for all dependent variables: strength use, job crafting, career development and job satisfaction. See Table 3 for regression results. Autism diagnosis was not a significant individual predictor for any of the dependent variables.

**Table 3.** Regression results.

	Job crafting			Use of strengths			Career development			Job satisfaction		
	β	SE	*t*	β	SE	*t*	β	SE	*t*	β	SE	*t*
Step 1	Adj. *R*^2^ = 0.47, *F*(2,187) = 12.55***			Adj. *R*^2^ = 0.07, *F*(2,187) = 8.60***			Adj. *R*^2^ = 0.22, *F*(2,187) = 27.09***			Adj. *R*^2^ = 0.11, *F*(2,187) = 12.47***		
(Constant)^ϯ^	50.23	5.16	9.73***	29.04	2.10	13.85***	5.76	1.27	4.47***	1.91	0.56	3.41***
Dx	−4.91	2.01	−2.45*	−1.84	0.81	−2.26	−0.98	0.49	−1.99	−0.32	0.22	−1.45
RWM	5.00	1.05	4.74***	1.63	0.43	3.83***	1.89	0.26	7.33***	0.51	0.11	4.45***
Step 2	Adj. *R*^2^ = 0.11, *F*(3,186) = 8.99			Adj. *R*^2^ = 0.07, *F*(3,186) = 5.85***			Adj. *R*^2^ = 0.11, *F*(3,186) = 8.99			Adj. *R*^2^ = 0.11, *F*(3,186) = 8.36***		
(Constant)	67.54	14.06	4.80***	32.41	5.73	5.66***	10.92	3.45	2.93***	2.58	1.53	1.69***
Dx	−18.02	10.11	−1.78	−4.39	4.12	−1.07	−4.34	2.48	−1.47	−0.20	1.10	−0.18
RWM	1.06	3.16	0.34	0.87	1.29	0.15	0.89	0.77	1.15	0.35	0.34	1.03
Dx x RWM	2.95	2.23	1.32	0.57	0.91	0.63	0.75	0.55	1.38	0.12	0.24	0.48

* *p* *<* 0.05;**p *<* 0.01; ***p *<* 0.001; ^ϯ^Unstandardised Beta reported.

After adding the interaction of autism diagnosis and RWM, none of the independent variables were significant predictors in any of the models. Furthermore, adding the interaction term did not significantly improve model fit explaining more of the variance than was previously attributed to individual independent variables. Thus, RWM was the single most important significant predictor of each dependent variable.

## Discussion

In the general population, a positive employee–manager relationship is associated with better career outcomes (Mumtaz & Rowley, 2020). This study investigated whether similar benefits are observed for autistic employees, and whether this differs from non-autistic adults. The findings indicate that one’s relationship with their manager, rather than whether one is autistic, is a significant predictor for: using one’s strengths at work, engaging in job crafting, access to career development opportunities and job satisfaction. Notably, the strength and direction of this association were consistent across both autistic and non-autistic employees. This suggests that for everyone, developing a positive relationship with one’s manager is an important factor in a range of work-related outcomes.

These findings align with the emerging research that identifies the importance of positive employee–manager relationships for autistic employees (Hayward et al., 2020; Martin et al., 2023). However, this study adds context by detailing how these relationships support the careers of autistic adults. For instance, the relationship with one’s manager is particularly important for access to career development opportunities, with 22% of the variance being explained by the regression model. For all the other dependent variables, only 7%–11% of the variance was explained. This indicates that there may be other factors not measured in this study that contribute to or are more important in predicting using one’s strengths at work, engaging in job crafting and job satisfaction: for example, personality type (H. Li et al., 2020), motivation (Aljumah, 2023; Lee & Song, 2020) and team dynamics (Lee & Song, 2020). These factors may also contribute to the quality of employee–manager relationships.

Given the importance of employee–manager relationships, it is concerning that autistic people rated their relationship with their manager as poorer than non-autistic participants. Although we asked participants to respond qualitatively to allow autistic participants to fully express themselves on their perception of the relationship with their manager, not enough detail was provided by participants to ascertain why the relationship was as they described. Indeed, we quantified rich qualitative data into a single-item measure, we did not use covariates and the effect is small. When we reviewed the other qualitative responses in the survey to identify any differing explanations, perceptions or experiences, we did not uncover any additional insights. Employing interactive methods in future research such as semi-structured interviews or focus groups might provide additional context and nuance.

Other theoretical lenses to examine employee–manager relationships, such as leader–member exchange theory, are also relevant (e.g. see works by Erdogan & Bauer, 2015; Lyubykh et al., 2020; Martin et al., 2023). Certainly, different mechanisms could be at play and need to be explored. For example, incongruence between employees and managers is associated with lower leader–member exchange (LMX) quality, which in turn negatively impacts work-related outcomes such as performance (Dwertmann & Boehm, 2016). Relational incongruence can also hinder inclusion at work (Longmire et al., 2025). This similarly relates to communication differences between autistic employees and non-autistic managers, and more specifically the double empathy issue (Williams et al., 2021). This notion refers to differences in processing, interpreting and interacting which contributes to mutual misunderstandings (Williams et al., 2021). The double empathy problem in the context of work has shown to result in reduced well-being and perceived lack of organisational support among autistic individuals (Hennekam & Follmer, in press). Communication differences, especially mutual misunderstandings, have been found to contribute to poorer employee–manager relationships, as seen in the general population (Fan & Han, 2018). Communication difficulties are more common for autistic employees (Hayward et al., 2018b, 2020). Autistic employees are often unfairly blamed for these misunderstandings, even when they stem from systemic factors (Bury et al., 2021).

Improving the relationship with one’s manager may boost job satisfaction, which was rated lower by autistic compared to non-autistic employees in the present study. The existing literature reports that job satisfaction is attributed to improved job performance (Inayat & Jahanzeb Khan, 2021; Prihadini, 2021) and employee retention (Htun & Bhaumik, 2022). Thus, we suggest that it is in all parties’ best interest to find ways to improve employee–manager relationships. Improved relationships might start with authentic leadership, which in turn has positive impacts for the employee and organisation, such as increased employee engagement (Baquero, 2023; Kleynhans et al., 2022). However, it is important that both employee and manager work towards establishing a positive relationship.

An unexpected finding of the present study was that no differences were found between participants on strengths use or job crafting measures. This may indicate good adaptive ability in the autistic sample, which may be indicated by later autism diagnoses (Tillmann et al., 2019). Most of our sample were diagnosed as autistic as adults at 25 years of age. Alternatively, given that the average age of participants was approximately 10 years since receiving their autism diagnosis at the time of completing the survey, it is also possible that our autistic participants understood their strengths to be able to apply them. The United Kingdom has many supports available to people post-autism diagnosis (e.g. see National Autistic Society).

### Limitations and suggestions for future research

The study’s sociocultural context, the United Kingdom, may also have contributed to participant’s ability to use their strengths and job craft. For example, recent changes in legislation allow UK employees to make a ‘statutory application’ (UK Government, 2023). Thus, employees can request flexible working arrangements from their first day of employment. Meaning that employees may have the flexibility they need to enhance their ability to use their strengths and participate in job crafting. Because there may be more job redesign or customisation at the outset of employment in this context, there may be less need for ongoing crafting. Given the influence of sociocultural and legal factors that vary from country to country, we recommend that future research explores cross-cultural comparisons with other countries. This could determine whether these findings are consistent or vary across different sociocultural settings and why this is the case. These insights can inform governmental policies to facilitate the working lives of autistic individuals.

It is increasingly acknowledged that the quality of the relationship between an autistic individual and one’s manager is of importance to create sustainable employment for this population (Martin et al., 2023). ‘Employment Autism’ in the United Kingdom has launched specific guidelines for managers to support autistic employees (Employment Autism, 2021). These include how to communicate and build a trusting relationship. Similarly, the National Autistic Society (UK) provides e-learning modules for employers to better understand and support autistic individuals in the workplace. Specialisterne, an agency specialised in neurodivergent workers, also provides concrete advice to managers regarding how to build rapport with autistic employees (Specialisterne, 2023). Thus, awareness of autism and workplace interpersonal supports are growing. Yet, to enhance feelings of belonging, more insights are needed to understand why the quality of the relationship between individuals with different neurotypes might be perceived to be lower (Longmire et al., 2025). What managers and co-workers can do to improve their relationships with autistic employees also requires further investigation (Longmire & Taylor, 2022).

It is acknowledged that one’s relationship with their manager is not static and might fluctuate or (d)evolve over time. In addition, it is short-sighted to consider that a single item might accurately capture this. These dynamics might be particularly salient given the unstable employment experiences reported by many autistic adults (Bury, Hedley, et al., 2024; Hayward et al., 2018a). To address this, future research could incorporate other methodological approaches, such as diary studies or longitudinal designs. These may capture the temporal variability in these relationships and provide a more comprehensive perspective.

We suggest a focus on qualitative methods because traditional quantitative surveys present several challenges for autistic individuals (Williams et al., 2021). This is largely due to differences in language processing and information needs (Williams et al., 2021). Ambiguous or vague wording can be problematic, as autistic people often interpret language literally and require greater precision to provide accurate responses (Wilson & Bishop, 2021). Forced-choice formats, such as Likert-type scales or binary options, may not allow for the nuanced and complex experiences that many autistic individuals wish to express (Stacey & Cage, 2023). In addition, lengthy or densely worded surveys can create significant cognitive load, leading to mental fatigue and potentially incomplete or rushed answers (Nicolaidis et al., 2020). Together, these can result in surveys that fail to capture the true breadth of autistic experiences.

The present study also only sought the perception of autistic and non-autistic employees. A more complete picture of the dynamic between employee and manager would be better understood by also capturing and comparing managers’ perceptions. This dyadic approach would provide greater clarity between employee and manager viewpoints enabling targeted recommendations. Furthermore, considering the nature of employee–manager relationships within a broader systems model (e.g. Bury, Zulla, et al., 2024) may help contextualise these findings.

It is important to acknowledge that other mechanisms may underlie career progression beyond those captured in the current study. For example, interpersonal liking between manager and employee may influence performance evaluations, potentially through halo effects (Thorndike, 1920), and subsequently impact career advancement. In addition, the use of same-source, cross-sectional data introduces the possibility of reverse causality. That is, access to career opportunities, the ability to use one’s strengths or engage in job crafting may not only be outcomes of a strong employee–manager relationship but could also shape employees’ perceptions of that relationship.

While an interaction term of autism diagnosis and RWM was included in the analysis, we stress that this result be interpreted with caution due to insufficient power to carry out this analysis robustly. Future research may wish to replicate our analysis with a larger sample or consider a path analysis. For example, to examine if a high-quality relationship with one’s manager is associated with job crafting and, in turn, the use of strengths which then explains career progress and job satisfaction.

Moreover, other concepts such as psychological safety and diversity climate may be important to include in future research endeavours, similarly suggested by Vogus and Taylor (2018). The former refers to the shared belief that it is safe to take an interpersonal risk without fear of punishment or reprisal (Edmondson, 1999). Diversity climate refers to employees’ shared perceptions of the degree to which an organisation utilises fair employee policies and socially integrates employees from underrepresented groups into the work setting (Mor Barak et al., 1998).

A final note on the sample in the present study is that they were generally well-educated people. This limits the generalisability of the findings beyond autistic individuals who do not have educational attainment beyond secondary school. Future research could investigate the relationships among autistic individuals with varying educational backgrounds to determine whether any significant differences exist. In a similar vein, most participants in our study received their autism diagnoses later in life, as adults. Their experiences may differ from autistic people who have been diagnosed early in life who may have been engaged in employment services throughout and after secondary schooling. It would be interesting to compare these groups of autistic individuals as doing so will yield important practical implications for early screening. In addition, it is interesting that the autistic employees in the present sample worked significantly more hours compared to the non-autistic sample. This contrasts with common assumptions and may represent an idiosyncrasy of our sample. As such, this finding highlights an opportunity for future research to explore the conditions and motivations underlying increased working hours among autistic individuals.

Finally, although the instruments used in the present study were not validated on an autistic population, Cronbach’s alpha was good to excellent. To the authors’ knowledge, there are no measures of this kind normed on an autistic population. Future projects may wish to consider other measures of employment outcomes which may provide broader context.

## Conclusion

This study highlights the importance of positive employee–manager relationships to sustained employment and career success, regardless of whether an employee is autistic. While the findings provide evidence about the equivalent importance of positive manager relationships on workplace behaviours and outcomes for autistic adults, they also highlight the social challenges faced by autistic employees. Autistic employees reported poorer relationships with their managers. Addressing these differences in relational outcomes requires organisations to better understand and support autistic employees, to ensure inclusive relationships with managers and supervisors. By advancing these efforts, managers can create environments that support the sustained and successful employment of autistic adults.
